# Zebrafish as animal model for aquaculture nutrition research

**DOI:** 10.3389/fgene.2014.00313

**Published:** 2014-09-10

**Authors:** Pilar E. Ulloa, Juan F. Medrano, Carmen G. Feijoo

**Affiliations:** ^1^Departamento de Ciencias Biologicas, Facultad de Ciencias Biologicas, Universidad Andres BelloSantiago, Chile; ^2^Department of Animal Science, University of California, Davis, Davis, CAUSA

**Keywords:** zebrafish, aquaculture nutrition, diet evaluation, nutritional genomics, nutritional immunity

## Abstract

The aquaculture industry continues to promote the diversification of ingredients used in aquafeed in order to achieve a more sustainable aquaculture production system. The evaluation of large numbers of diets in aquaculture species is costly and requires time-consuming trials in some species. In contrast, zebrafish (*Danio rerio*) can solve these drawbacks as an experimental model, and represents an ideal organism to carry out preliminary evaluation of diets. In addition, zebrafish has a sequenced genome allowing the efficient utilization of new technologies, such as RNA-sequencing and genotyping platforms to study the molecular mechanisms that underlie the organism’s response to nutrients. Also, biotechnological tools like transgenic lines with fluorescently labeled neutrophils that allow the evaluation of the immune response *in vivo*, are readily available in this species. Thus, zebrafish provides an attractive platform for testing many ingredients to select those with the highest potential of success in aquaculture. In this perspective article aspects related to diet evaluation in which zebrafish can make important contributions to nutritional genomics and nutritional immunity are discussed.

## AQUACULTURE NUTRITION: TRENDS AND FUTURE PERSPECTIVE

Worldwide fish production is increasing at 8.8% per year (Food and Agriculture Organization of the United Nations [FAO], 2012). However, the decreasing availability of fishmeal because of a reduction in the supply of important sources of fish limits its use as the primary protein in fish diets. In fact, harvest volumes of these species decreased from 10.7 million tons in 2004 to 4.2 million tons in 2010 (Food and Agriculture Organization of the United Nations [FAO], 2012). This outcome, apart to generate a negative ecological impact, has led to an increase in the value of fishmeal, affecting the profitability of many aquaculture enterprises (Rosamond et al., 2000). Therefore, the utilization of plant protein meals has emerged as an alternative to replace of fish meal in aquaculture feeds (Hardy, 2010).

Within the wide range of available vegetable protein sources (peas, lupine, maize, and wheat), soybean has been the most commonly used due to its wide availability in the market, low cost, high content of digestible protein and balanced amino acid profile (Naylor et al., 2009). Increasing dietary levels of soybean meal and other vegetable proteins has been tested in a variety of fish species, with inclusion levels ranging from 20 to 100% of fishmeal replacement. Unfortunately, results have shown several adverse effects such as reduction of growth and intestinal inflammation, even at low levels of inclusion (Gómez-Requeni et al., 2004; Mundheim et al., 2004; Knudsen et al., 2007; Urán et al., 2008).

Different approaches have been taken to make the utilization of plant protein by different fish more efficient, including the improvement of genetic selection in plants aiming to reduce the effect of anti-nutritional components and the stimulation of the intestinal microbiota of fish (Bakke-McKellep et al., 2007; Froystad-Saugen et al., 2009; Merrifield et al., 2009). Additionally, the diversification of new protein ingredients (from animals and plants), and identification of additives (natural or synthetic) with “intestinal protective” activity to promote growth and health have been made a priority (Refstie et al., 2010; Sicuro et al., 2010; Czubinski et al., 2014). Thus, the effects of nutrition on genomics and immunity are being addressed (Montero et al., 2010; Hernández et al., 2013). The implementation of new technologies such as RNA-sequencing, together with progress in sequencing genomes of different fish, can identify the genes affected by nutrition and also identify the genetic variants that influence the organism’s response to nutrients.

To fully understand the repercussions of new diets on fish physiology, a shift in approach is requires to determine the molecular and cellular pathways that regulate responses to different diets. The evaluation of a large numbers of diets directly in aquaculture species results in high costs and long-term assays, so it is necessary to implement new strategies in order to accelerate and make this experimental process cost-effective. It is also essential to determine the molecular mechanisms by which physiological process are perturbed in response to diet. This will provide insights on how to solve existing problems resulting from nutrition interventions in the aquaculture industry.

As an alternative approach to addresses the aforementioned issues, we propose the use of zebrafish (*Danio rerio*) as an animal model for high throughput testing of experimental diets in aquaculture. This teleost fish has numerous advantages related to its fast life cycle, ease of handling and very well-known biology, besides allowing *in vivo* analysis with large numbers of fish (Kimmel et al., 1995). Here, we highlight the important contribution that zebrafish can make in the field of nutritional genomics and nutritional immunity. Both lines of investigation provide useful contributions to the evaluation of diets.

## ADVANTAGES OF ZEBRAFISH AS AN ANIMAL MODEL FOR AQUACULTURE NUTRITION RESEARCH

Zebrafish is a well-established animal model for a wide range of research areas, from biomedicine to toxicology (Roush, 1996; Bergeron et al., 2008). The use of this model fish for improving production process of aquaculture has emerged as an important research field (Ulloa et al., 2011; Ribas and Piferrer, 2013). In particular, research improving husbandry and survival, immune response, nutrition and growth have been carried out in zebrafish, and are expected to provide results applicable to important commercial fish (Oyarbide et al., 2012; Hedrera et al., 2013; Ulloa et al., 2013).

Among the advantages of this model are its ease of handling in breeding and experimentation, short generation intervals (∼3 months) and large numbers of eggs per brood (100–200 eggs/clutch), which allow performing all analyses with a high number of specimens per data point (Kimmel et al., 1995). Embryos hatch at 2 days post-fertilization and larvae can live for 5 days without feeding due to the consumption of the yolk (Lawrence, 2007). During the larval period all organs and systems are functional, making these individuals physiologically equivalent to adult animals. In fact, both larvae and adult zebrafish can eat a wide variety of foods including live feed (*Paramecium* and *Artemia* nauplii) and different commercial fish diets, as well as experimental plant protein-based diets (Hedrera et al., 2013; Ulloa et al., 2013). The availability of a sequenced genome (assembly ZV9) allows evaluating the effect of diet on molecular mechanisms using genomic tools such as RNA-sequencing (RNA-seq; Morozova and Marra, 2008). This technology has been recently used in some aquaculture species and also in zebrafish (Li et al., 2013; Long et al., 2013; Xu et al., 2013; Cui et al., 2014; Liu et al., 2014). On the other hand, a wide diversity of approaches in genetic manipulation are readily available in zebrafish. The availability of a large number of transgenic lines, which carry specific promoters coupled to GFP (green fluorescent protein), is widespread. For example, the use of the Tg(Bacmpx:GFP) line allows to follow specific innate immune cells such as neutrophils *in vivo* due to its fluorescent mark (Renshaw et al., 2006). Moreover, it has been demonstrated that neutrophils output from hematopoietic tissue toward affected territories correlates with pro-inflammatory cytokine production, thus making transgenic lines “live indicators” of inflammatory process (Barros-Becker et al., 2012). All these assays can be carried out with embryos and larvae, which are distributed individually or in small groups in micro well plates in small volumes (0.5–2 ml) allowing sufficient biological replicas in each experiment.

Directly related to the evaluation of diets are two aspects in which zebrafish can make important contributions: nutritional genomics and nutritional immunity.

## NUTRITIONAL GENOMICS CONTRIBUTIONS

Nutritional genomics is a discipline that investigates the relationship between the genome and diets. Two approaches are essentially used: “Nutrigenomics,” which studies how dietary ingredients affect the gene expression and “Nutrigenetics,” which aims to understand how the genetic makeup of an individual coordinates the response to diet (Mutch et al., 2005). Both approaches attempt to clarify the effect of dietary components that contribute to phenotype by altering gene expression and individual genetic variants.

Since one effect of plant diets on fish phenotypes is growth, for more than one decade two approaches have been used to understand the genomics associated with fish growth: (1) global evaluation of genes by the creation of microarray platforms based on EST libraries, and (2) evaluation of candidate genes involved in growth. These studies have generated a list of genes that are over/under expressed in response to vegetable diets in different development stages of fish (Douglas, 2006; Panserat et al., 2008; Von Schalburg et al., 2008; Alami-Durante et al., 2010; Tacchi et al., 2011). However, the results obtained in the many nutritional studies are commonly difficult to compare among each other. This is due to the use of different origins of the same ingredient, feed formulation, genetic background of fish and experimental design. Moreover it is a common practice that, the biological samples used for transcription analysis are randomly selected. This experimental background makes interpretation of data difficult in order to dissect the relationship between genotype and phenotype, as well as the effects of diet on gene expression. Thus, in order develop a better understanding of molecular mechanism modulated by nutrition, it is necessary to select fish according to genotype, phenotype and/or ideally based on genetically improved populations (Kolditz et al., 2008; Morais et al., 2011; Salem et al., 2012).

Advanced technologies such as RNA-seq and genotyping platforms allow accelerated research in the nutritional genomics field that can be projected to aquaculture species (Houston et al., 2014; Qian et al., 2014). These technologies have been widely used to increase the genomic understanding of phenotypes in other livestock species (Wickramasinghe et al., 2014). A recent aquaculture study analyzed genotype-diet interaction in the transcriptome analysis of Atlantic salmon fed vegetable oil. The authors identified metabolic pathways and key regulators that may respond differently to alternative plant-based feeds depending on genotype (Morais et al., 2011). Salem et al. (2012) using RNA-seq, identified 23 single nucleotide polymorphisms (SNPs) markers in rainbow trout associate with growth response to commercial fish meal-based diet. However, despite these efforts, the identification of genetic differences (gene expression and SNPs) among fish in relation to growth rates in response to plant protein diets has not been reported.

In order to address this subject, a new approach using zebrafish was developed (Ulloa et al., 2013). Briefly, a population of 24 experimental families was generated to examine growth response in zebrafish fed with a plant protein-based diet. At 30 days post-fertilization (dpf) each family was split to generate two replicates (40 fish per family replicate), which created two populations of fish with similar genetic backgrounds. The fish were fed from larval transition (35 dpf) to sexual maturity (98 dpf). The first replicate of 24 families was fed a diet containing 100% plant protein as the only protein source (experimental diet) and the second replicate was fed a diet containing 100% animal protein as the only protein source (control diet). The results showed decreased growth in fish fed a plant protein-based diet compared to fish fed a fish meal-based diet, as has been documented in farmed fish (Gómez-Requeni et al., 2004; Mundheim et al., 2004), and very large growth variations from juvenile to adult stages among families (Ulloa et al., 2013). In order to evaluate the effect of a plant protein-based diet on the expression of growth-related genes in the muscle of zebrafish, individuals from three similar families representative of the mean weight in both populations were selected. To understand the effect of family variation on gene expression, these families were evaluated separately. The results showed that the effect of plant diet and family variation on gene expression were significantly different, and clearly suggested that gene expression is influenced not only by nutrition but also by genetic differences in each family; such as been described by Gjedrem (2000). Thus, it was demonstrated that to understand the effect of diet on transcriptome analysis, it is important to homogenize the phenotype and genetic components to avoid conflicts in the interpretation of results (Ulloa et al., 2013).

To measure gene expression and identify SNPs to evaluate growth associations in zebrafish fed a plant protein diet, a new approach was developed using RNA-seq. Samples of muscle collected from low and high growth fish were analyzed to identify SNP in differentially expressed genes. One hundred twenty-four genes were differentially expressed between phenotypes. From these genes 164 SNP were selected and genotyped in 240 fish samples. Marker-trait analysis revealed five SNP in key genes directly related with growth response (Unpublished data). This study provided new candidate genes associated with growth that could be evaluated in farmed fish through comparative genomics. Additionally, this strategy promises to be useful to identify SNP and characterize individuals with the highest performance for growth in response to a plant protein-based diet.

## NUTRITIONAL IMMUNITY CONTRIBUTIONS

Diverse studies in cultured fish have shown that soybean meal induces intestinal inflammation, a pathology called enteritis (Bakke-Mckellep et al., 2000; Knudsen et al., 2007). The hallmark of innate immunity is inflammation, this process is triggered in response to different insults, including pathogens, injury or irritants (Chen and Nuñez, 2010). When inflammation occurs, influx, accumulation, and activation of leukocytes (predominantly neutrophils) are triggered at the affected site during the early stages of the response (Witko-Sarsat et al., 2000). One of the first cytokine to be released when inflammation occurs is the pro-inflammatory cytokines Interleukin-1 β (IL-1β; Rodriguez-Tovar et al., 2011). Other essential proteins for neutrophils chemoattraction and migration are the chemokine Cxcl8 and some metalloproteinase enzymes (MMPs). Cxcl8 promotes neutrophils recruitment to the sites of insult; meanwhile MMPs are involved in the degradation of the extracellular matrix in order to promote granulocytes migration (Oliveira et al., 2013). Once neutrophils reach the affected site, they destroy the *insulting agent* through the production of non-specific toxins (Fialkow et al., 2007).

On the opposite side are the anti-inflammatory cytokines, such as transforming growth factor beta (TGF-β) and Interleukin 10 (Il-10), which are mainly secreted by macrophages when the inflammatory agent is removed, promoting the end of the inflammatory process (Engelsma et al., 2002; Ouyang et al., 2011). It is noteworthy that an inflamed intestine of fish is characterized by shorter mucosal folds, loss of vacuole absorptive cells in the intestinal epithelium and large infiltration of neutrophils, macrophages and, eosinophils in the *lamina propria*, among others (Baeverfjord and Krogdahl, 1996).

The severity of inflammation differs between species and depends on the percentage of plant feeds inclusion to the diets. In salmonids, the most affected by the inclusion of plant protein appears to be Atlantic salmon (*Salmo salar*) and to a lesser extent rainbow trout (*Oncorhynchus mykiss*; Morris et al., 2005; Bakke-McKellep et al., 2007). However, the effect of inflammation has also been described in omnivorous fish such as carp (*Cyprinus carpio*) and zebrafish (Urán et al., 2008; Hedrera et al., 2013). This situation affects the cellular and humoral immunological processes, with negative consequences in food intake and growth (Gómez-Requeni et al., 2004; Mundheim et al., 2004; Montero et al., 2010).

In recent years, additives such as prebiotics mannooligosaccharides (MOS) and fructoligosaccharides (FOS), probiotics (bacteria); immunostimulants (β-glucans), and nucleotides have been incorporated into fish diets in order to control diseases, improve health, and the immune status against acute stress (Piaget et al., 2007; Staykov et al., 2007; Tahmasebi-Kohyani et al., 2012). In the case of MOS, the supplementation of 0.2% into diets with 14% inclusion of soybean meal decreased the intestinal inflammation in Atlantic salmon (Refstie et al., 2010). In sea bream, the effect of supplementation of 0.4% MOS into diets with 31% inclusion of soybean meal revealed an increase of microvilli density and length of intestinal folds (Dimitroglou et al., 2010). These results showed that MOS have a protective effect on intestinal inflammation triggered by soybean meal. However, multiple factors were involved, such as interspecific variation, inclusion of soybean meal and percentage of additive used in the supplementation. Thus, further research is needed to compare efficiencies between new “intestinal protector” additives.

To develop research in zebrafish to find solutions to the issues mentioned above requires first the corroboration that intestinal inflammation triggered by soybean meal in zebrafish recapitulates what is seen on farmed fish. This approach has been addressed exactly by Hedrera et al. (2013). They present a new strategy to analyze the potential intestinal impact that the intake of different food ingredients can trigger. Specifically, they analyzed the effects of the ingestion of soybean meal and two of its components, soy protein and soy saponin in zebrafish. Demonstrating that larvae fed with soybean meal developed an intestinal inflammation as early as 2 days after start feeding. Moreover, it was observed that saponin but not soy protein extract was responsible for the inflammatory response.

**FIGURE 1:**
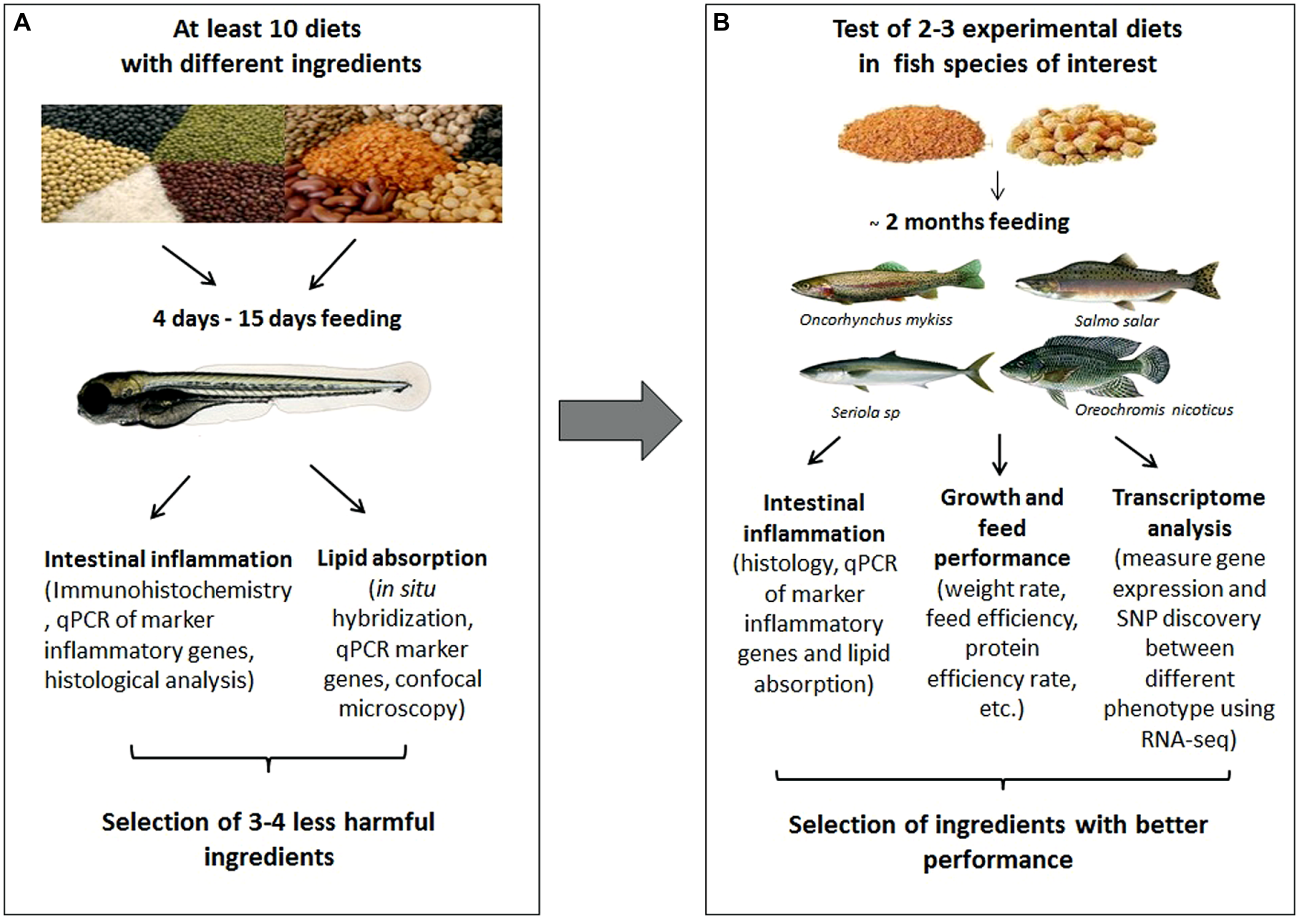
**Diagram showing a proposed strategy using zebrafish to evaluate ingredients with potential use in aquaculture nutrition.** **(A)** Diet pre-selection step in zebrafish. Different biotechnological tools available in zebrafish allow a detail physiological analysis to evaluate many ingredients and select the less harmful or more beneficial. **(B)** Selection step in the aquaculture fish species. The diets can be tested in the fish species of interest to confirm the benefits from the pre-selected ingredients in zebrafish. Finally, the best diets could be incorporated to an aquaculture nutrition program.

These findings support the use of zebrafish screening assays to identify novel ingredients/additives that would lead to improved current fish diets or to the formulation of new ones. **Figure 1** illustrates the two steps in the new proposed strategy. The first step is a “pre-screening” developed in zebrafish. The aim of this step is to evaluate a large number and wide range of ingredients or additives in order to select the more beneficial or less harmful. The second step considers the determination of the intestinal effects generated by the selected ingredients on the target fish specie. This method eliminates the need to evaluate all diets directly on commercial fish, reducing high costs and time consuming experimentation.

Besides zebrafish has potential in nutritional studies, it is important to highlight that assays in this fish cannot replace analysis in farmed fish, as well as results cannot be direct extrapolated to other fish species. For example, results regarding the level of enteritis triggered by soybean meal in Atlantic salmon are different from those detected in rainbow trout. What is important is that in both species soybean causes intestinal inflammation that is mainly due to the saponin, which also occurs in zebrafish. Moreover, in all these species the level of proinflammatory cytokines are upregulated, suggesting that the molecular mechanisms are conserved. In a similar way, several studies have shown that the intake of a diet based on soybean meal decreases the growth rate of salmon, rainbow trout, carp, tilapia, sea bream, and also zebrafish (Pongmaneerat et al., 1993; Médale et al., 1998; Fontaínhas-Fernandes et al., 1999; Gómez-Requeni et al., 2004; Mundheim et al., 2004; Ulloa et al., 2013). These results suggest that the biological processes and molecular mechanisms that underlie the growth response to nutrients overlap among different fish, regardless of evolutionary distance or environmental conditions. Understanding how signaling cascades are coordinated and their effects on physiological response, such as growth and inflammation, may be unraveled in zebrafish. Thus, investigations undertaken in zebrafish nutrition could make important contributions to aquaculture nutrition research.

## FUTURE DIRECTIONS

The current challenge is to apply the knowledge obtained in zebrafish to benefit the aquaculture industry. In the future, one of the principal challenges will be to cultivate carnivorous fish that can tolerate higher levels of plant protein in their diet. New technologies such as RNA-seq and genotyping platforms will be key in our ability to select fish with increased tolerance to a vegetal protein diet. Identification of more friendly vegetal ingredients should also be examined. Thus, it is not hard to imagine that in a near future fish diets will be formulated with ingredients and/or additives according to the genetic background of the strain of interest instead of depending solely on the species.

## AUTHOR CONTRIBUTIONS

Pilar E. Ulloa: drafting of the manuscript; Carmen G. Feijoo: drafting of the manuscript and critical revision of the intellectual content; Juan F. Medrano: critical revisions of the intellectual content.

## Conflict of Interest Statement

The authors declare that the research was conducted in the absence of any commercial or financial relationships that could be construed as a potential conflict of interest.
